# Optimal timing of renal replacement therapy initiation in acute kidney injury: the elephant felt by the blindmen?

**DOI:** 10.1186/s13054-017-1713-2

**Published:** 2017-06-20

**Authors:** Chih-Chung Shiao, Tao-Min Huang, Herbert D. Spapen, Patrick M. Honore, Vin-Cent Wu

**Affiliations:** 1 Division of Nephrology, Department of Internal Medicine, Saint Mary’s Hospital Luodong, No. 160 Chong-Cheng South Road, Loudong 265, Yilan, Taiwan (Republic of China); 2 Saint Mary’s Junior College of Medicine, Nursing and Management, No.100, Ln. 265, Sec. 2, Sanxing Road, Sanxing Township, Yilan County, 266 Taiwan (Republic of China); 3 Division of Nephrology, Department of Internal Medicine, National Taiwan University Hospital, No. 7 Chung-Shan South Road, Zhong-Zheng District, Taipei, 100 Taiwan (Republic of China); 4 ICU Department, Universitair Ziekenhuis Brussel, Vrije Universiteit Brussel, 101, Laarbeeklaan, 1090 Jette, Brussels, Belgium; 5 NSARF, National Taiwan University Study Group on Acute Renal Failure, Taipei, Taiwan (Republic of China)

**Keywords:** Acute kidney injury, Delayed, Early, Intensive care unit, Renal replacement therapy, Timing

## Abstract

Renal replacement therapy (RRT) is a key component in the management of severe acute kidney injury (AKI) in critically ill patients. Many cohort studies, meta-analyses, and two recent large randomized prospective trials which evaluated the relationship between the timing of RRT initiation and patient outcome remain inconclusive due to substantial differences in study design, patient population, AKI definition, and RRT indication. A cause-specific diagnosis of AKI based on current staging criteria plus a sensitive biomarker (panel) that allows creating a homogeneous study population is definitely needed to assess the impact of early versus late initiation of RRT on patient outcome.

## Background

Acute kidney injury (AKI) is a common yet highly devastating complication in critically ill patients [1]. AKI is associated with increased morbidity, mortality, and healthcare costs [2]. Renal replacement therapy (RRT) remains a cornerstone of AKI treatment in the intensive care unit (ICU). However, RRT is a double-edged “therapeutic” sword, in particular with regard to timing of intervention [3]. Early initiation may control fluid and electrolyte status more efficiently, more rapidly correct acid–base homeostasis, remove uremic toxins appropriately, and perhaps prevent subsequent complications attributable to AKI [4]. RRT initiated before the onset of severe AKI could potentially prevent the kidney-specific damage and remote organ injury resulting from fluid overload, electrolyte–metabolic imbalance, and systemic inflammation. However, early initiation of RRT may also unnecessarily expose patients, who might recover from AKI without RRT, to unwarranted complications associated with RRT use. These complications include hemodynamic instability, coagulation disorders, bloodstream infection, and even inflammatory or oxidative stress induced by bio-incompatibility reactions to dialyzer membranes [5]. Late initiation of RRT may provide time to stabilize the patient’s condition or more adequately treat underlying diseases so that unnecessary renal support is avoided [6]. However, acting too late holds a potential risk of delaying crucial therapy and may worsen prognosis.

## The timing of RRT initiation and outcome: an elephant touched by blind men?

Seabra et al. analyzed 23 studies including five randomized controlled trials (RCTs) and reported a significant survival benefit when RRT was started early. The observed benefit was predominantly found in cohort studies but was not confirmed in the RCTs [7]. Karvellas et al. conducted a meta-analysis of 13 observational studies and two small RCTs. They also demonstrated a significant benefit in 28-day survival in the early RRT group [8]. In contrast, an extensive evidence-based systematic review enrolling the most recently published studies concluded that early RRT did not improve patient survival or confer reductions in ICU or hospital length of stay [9]. These incongruous results are due to differences in study quality, publication bias, heterogeneous patient populations (e.g. medical vs surgical patients), various AKI definitions and subtypes, and different cutoff points at which clinicians decide to start RRT (e.g., urine output, metabolic variables, AKI severity, or temporal relationship with particular events) [8–10].

AKI definitions which are based essentially on the measurement of urinary output and serum creatinine levels have been refined progressively for diagnostic, prognostic, and research purposes. Expert panels have successively proposed the Risk, Injury, Failure, Loss, and End-stage (RIFLE) renal disease criteria in 2004, [11] the AKI Network (AKIN) criteria in 2007 [12], and the Kidney Disease Improving Global Outcomes (KDIGO) AKI criteria in 2012 [13]. Studies that applied these RIFLE, AKIN, or KDIGO criteria to evaluate patient outcomes related to the early or late timing of RRT initiation are summarized in Tables 1 and 2 [14–23]. Observational studies demonstrate better outcome in patients receiving early RRT treatment but this is not confirmed in RCTs [14–23]. Of note is that many studies are retrospective or prone to a type I error in hypothesis testing due to significant differences in preintervention study groups [9].

**Table 1:** Summary of studies using RIFLE, AKIN, and KDIGO criteria for outcome evaluation

Author (year)	ICU setting	RRT modality	Inclusion criteria	Exclusion criteria	*n*	End points
Randomized controlled trials						
Zarbock 2016 [24]	Predominantly surgical	CVVH	KDIGO stage 2 AKI	eGFR < 30 ml/min/1.73 m^2^, previous RRT, AKI caused by permanent occlusion of renal artery or surgery, GN, IN, HUS, AIDS, HRS, pregnancy	231	30-day, 60-day and 90-day mortality
Gaudry 2016 [23]	Mixed	Mixed	Ischemic or toxic AKI and receiving MV, catecholamine infusion or both, and KDIGO stage 3 AKI	BUN > 112 mg/dl, sK > 6.0 mmol/L, pH < 7.15, acute pulmonary edema	619	30-day and 60-day mortality
Prospective cohort studies						
Sabater 2009 [14]	Medical	CVVH	N/A	N/A	148	In-hospital mortality
Shiao 2009 [15]	Surgical	CRRT/SLED/IHD	Postoperative AKI requiring RRT in ICU (s/p major abdominal surgery)	Age < 18 years; ICU stay < 2 days; RRT started before surgery; no abdominal surgery; renal transplant	98	In-hospital mortality
Retrospective cohort studies						
Chou 2011 [16]	Medical	CRRT/ SLED	Septic AKI s/p acute RRT	Age <18 years; ICU stay < 2 days; RRT < 2 days	370	In-hospital mortality
Wu 2012 [17]	Surgical	CRRT	(1) AKI with sK > 6.0 meq/L, (2) metabolic acidosis (sHCO_3_ < 12 meq/L), (3) pulmonary edema refractory to diuretics, or (4) oliguria with progressive azotemia, especially in hemodynamically unstable patients	N/A	73	60-day and 90-day mortality
Boussekey 2012 [18]	Mixed	N/A	ICU patients in need of RRT	N/A	110	In-hospital mortality
Hu 2013 [19]	Mixed	CRRT	AKI with CRRT	CKD	52	In-hospital mortality
Shum 2013 [20]	Medical	CRRT	Septic AKI	Cardiothoracic surgery, transplant surgery, and burns	120	In-hospital mortality
Leite 2013 [21]	Mixed	SLED	ICU patients on acute RRT	CKD	150	In-hospital mortality
Suzuki 2013 [22]	Mixed	CRRT	AKI with CRRT	N/A	189	In-hospital mortality

This original table was created by the authors

*AIDS* acquired immune deficiency syndrome, *AKI* acute kidney injury, *AKIN* Acute Kidney Injury Network, *BUN* blood urea nitrogen, *CKD* chronic kidney disease, *CRRT* continuous renal replacement therapy, *CVVH* continuous venovenous hemofiltration, *eGFR* estimated glomerular filtration rate, *GN* glomerular nephritis, *HRS* hepatorenal syndrome, *HUS* hemolytic uremic syndrome, *ICU* intensive care unit, *IHD* intermittent hemodialysis, *IN* interstitial nephritis, *KDIGO* Kidney Disease Improving Global Outcomes, *MV* mechanical ventilation, *N/A* not applicable, *RRT* renal replacement therapy, *sK* serum potassium, *SLED* sustained low-efficiency dialysis

**Table 2:**
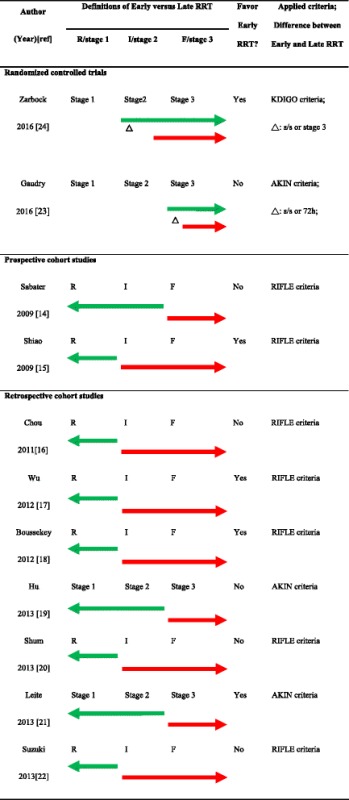
Cutoff points and outcomes of early versus late RRT initiation

This original table was created by the authors. Coverage of the arrows illustrates the cutoff points and definitions of early (*green*) versus late (*red*) RRT initiation

*AKIN* Acute Kidney Injury Network, *KDIGO* Kidney Disease Improving Global Outcomes, *RIFLE* Risk, Injury, Failure, Loss, and End-stage renal disease, *RRT* renal replacement therapy

## The AKIKI and ELAIN trials: any solace?

Two recently published large prospective RCTs, the Artificial Kidney Initiation in Kidney Injury (AKIKI) trial [23] and the Early versus Late Initiation of Renal Replacement Therapy in Critically Ill Patients with Acute Kidney Injury (ELAIN) trial [24], have assessed the impact of different RRT timing in severely ill ICU patients with AKI without potentially life-threatening complications. The AKIKI and ELAIN trial concepts are outlined in Table 3. The AKIKI trial [23] enrolled 620 ICU patients on mechanical ventilation and/or catecholamine infusion with KDIGO stage 3 AKI. No significant difference in 60-day mortality was found between early and delayed RRT. The ELAIN trial [24] included 231 ICU patients with KDIGO stage 2 AKI and exhibiting a plasma neutrophil gelatinase-associated lipocalin (NGAL) level above 150 ng/ml. Compared with delayed treatment, an early strategy resulted in lower 90-day mortality, more rapid recovery of renal function, and a significantly shorter duration of hospital stay.

**Table 3:** Comparison of the AKIKI and ELAIN trials

	AKIKI trial [23]	ELAIN trial [24]
Study design	Multicenter (31 ICUs in France): randomized, unblinded	Single center (one ICU in Germany): randomized, unblinded
Patient characteristics and number	Predominantly medical patients (79%); *n* = 620 (from 5528 screened patients (11%))	Predominantly postsurgical patients (97%); *n* = 231 (from 604 screened patients (38%))
Age at enrollment (years)	66.1^a^	67.0^a^
SOFA score at enrollment	10.9^a^	15.8^a^
Septic shock at enrollment (%)	66.7	32.0
Enrollment criteria	ICU patients, ≧18 years old; KDIGO stage 3 AKI; at least one of the following: MV, catecholamine need	ICU patients, 18–90 years old; KDIGO stage 2 AKI; plasma NGAL > 150 ng/ml; at least one of the following: severe sepsis, catecholamine need, nonrenal organ dysfunction, fluid overload
Criteria for RRT in EG	KDIGO stage 3 AKI (within 6 h)	KDIGO stage 2 AKI (within 8 h)
Criteria for RRT in DG	Any of the following: BUN > 112 mg/dl, sK > 6 mEq/L, pH < 7.15, lung edema, oliguria > 72 h	KDIGO stage 3 AKI or any of the following (within 12 h): BUN > 100 mg/dl, sK > 6 mEq/L, sMg > 8 mEq/L, organ edema, U/O < 200 ml/h
SCr at RRT (mg/dl)	3.3 (EG) vs 5.3 (DG)^a^	1.9 (EG) vs 2.4 (DG)^a^
Time to RRT (h)	2.0 (EG) vs 57.0 (DG)^b, c^	6.0 (EG) vs 25.5(DG)^b, d^
Initial modality	55.0% IHD, 45.0% CRRT (modality not available)	100.0% CRRT (CVVHDF)
Receipt of RRT	EG (98.0%) > DG (51.0%) (*p* < 0.001)	EG (100.0%) > DG (91.0%) (*p* < 0.001)
Primary endpoint	60-day mortality EG (48.5%) ≒ DG (49.7%) (*p* = 0.79)	90-day mortality EG (39.3%) < DG (54.7%) (*p* = 0.03) → EG better
Other outcomes	Catheter-related-infection: EG (10.0%) > DG (5.0%) (*p* = 0.03) → DG better	Median LOS: EG (51 days) < DG (82 days) (*p* < 0.001) Duration of MV: EG (126 h) < DG (181 h) (*p* = 0.002) → EG better
Special remarks	60-day mortality: all EG (48.5%) ≒ DG (49.7%); DG/RRT(–) (37.1%) < EG (48.5%) < DG/RRT(+) (61.8%)	

This original table was created by the authors

^a^Mean value

^b^Median value

^c^“From randomization to RRT initiation”

^d^“From meeting eligibility criteria to RRT initiation”

*AKI* acute kidney injury, *AKIKI* Artificial Kidney Initiation in Kidney Injury, *BUN* blood urea nitrogen, *CRRT* continuous renal replacement therapy, *CVVHDF* continuous venovenous hemodiafiltration, *DG* delayed treatment group, *EG* early treatment group, *ELAIN* Early versus Late Initiation of Renal Replacement Therapy in Critically Ill Patients with Acute Kidney Injury, *h* hour(s), *ICU* intensive care unit, *IHD* intermittent hemodialysis, *KDIGO* Kidney Disease Improving Global Outcomes, *LOS* length of stay, *MV* mechanical ventilation, *NGAL* neutrophil gelatinase-associated lipocalin, *pH* potential of hydrogen, *SCr* serum creatinine, *sK* serum potassium, *sMg* serum magnesium, *SOFA* Sequential Organ Failure Assessment, *RRT* renal replacement therapy, *U/O* urine output

The discrepant outcome result between both trials is confusing but can be explained by important methodological differences. First, the AKIKI trial was conducted in 31 ICUs screening 5528 predominantly medical patients for 25 months to finally randomize 620 (11%) subjects. The ELAIN trial was a single-center trial conducted over a similar time period but screening only 604 almost exclusively postsurgical and trauma patients to include 231 (38%) subjects. This suggests potential patient selection, inclusion, and treatment bias. Second, patients in the ELAIN trial received delayed RRT more “early” than their AKIKI counterparts (25.5 h vs 57 h). The modest difference in RRT initiation time in the ELAIN trial is also difficult to reconcile with the observed positive effects on outcome. Third, both trials included patients with different disease severity and AKI etiology. Patients with refractory pulmonary edema were excluded in the AKIKI trial but accounted for three-quarters of ELAIN inclusions. ELAIN patients had more nonrenal organ dysfunction (as shown by a higher baseline Sequential Organ failure Assessment score at enrollment). Also, septic AKI which was more prevalent in AKIKI patients and postoperative AKI have different pathophysiology and prognosis. Fourth, according to the applied AKI definition, patients entering the AKIKI trial all had at least “renal failure” (KDIGO stage 3 AKI) whereas this was only the case for the delayed ELAIN treatment group. Patients receiving early treatment in the ELAIN trial were thus included with “less severe” AKI, which could have beneficially influenced outcome. Fifth, initial RRT modalities were at the discretion of the enrolling AKIKI investigators which resulted in a mix of continuous and intermittent RRT techniques. In contrast, all patients in the ELAIN trial were started on continuous venovenous hemodiafiltration and the majority was transitioned to daily sustained low-efficiency dialysis. The latter technique was never employed in AKIKI patients. Differences in fluid and metabolic dynamics between various RRT modalities may have determined hemodynamic assessment, treatment, and outcome in a substantial number of patients. Finally, up to half of the patients allotted to late treatment in the AKIKI trial ultimately did not receive RRT. This cohort had the lowest mortality rate (37.1%) as compared with patients receiving either early (48.5%) or late (61.8%) RRT. Despite adjustment for baseline severity of illness, the impact of protocol-associated patient selection and protocol-mandated delay in RRT on outcome should be considered [25, 26].

## STARRT-AKI trial: another touch of the elephant?

Besides the two aforementioned RCTs, another ongoing large multinational, multicenter RCT, the “STandard Versus Accelerated Initiation of Renal Replacement Therapy in Acute Kidney Injury (STARRT-AKI)” trial, deserves attention. The STARRT-AKI trial aims to enroll a large number of patients worldwide (2866 subjects in more than 60 sites across countries) and thus is expected to be more representative than the AKIKI and ELAIN trials. Moreover, the choice for early or delayed initiation of RRT in this trial will be determined by a “KDIGO stage 2” or by “specific clinical criteria” respectively, which more closely reflects current ICU practice [27]. Although plasma NGAL has low indicating power for estimating the possibility of AKI progression or the optimal timing for RRT initiation [27], the fact that no biomarker is selected for screening or risk stratification purposes might be a potential shortcoming of this trial.

## Practical implications and prospects: we plea for a universal AKI definition!

AKI is a complex disorder with many potential (i.e., septic, ischemic, or toxic) triggers. Prerenal, intrarenal, and postrenal disorders may either alone or in combination contribute to AKI severity and progression [28]. All of these factors finally will determine patient outcome. On top of this, RRT is increasingly implemented in the treatment of AKI, even in the absence of life-threatening hemodynamic or metabolic conditions. Basing decisions on creatinine concentrations or urinary output is unreliable in critically ill ICU patients. Moreover, the prognosis may also vary in patients who are diagnosed with similar AKI stage at different time points (e.g., at admission or during hospitalization) [28, 29]. Thus, currently applied AKI criteria should be adapted and perhaps strengthened by adding sensitive functional and structural biomarkers [28, 29].

Several novel biomarkers have been introduced as an aid to identify patients with AKI earlier, to evaluate severity of kidney injury, to differentiate type and etiology of injury, and to assess the effect of interventions on renal recovery [30, 31]. Some biomarkers may even independently detect AKI progression regardless of glomerular filtration rate changes [32]. Actual biomarkers lack specificity for correctly assessing the time of AKI occurrence but are useful for risk stratification in severe AKI and for determining the need for RRT or mortality prediction [30, 33]. Furthermore, a clinical approach supported by biomarker assessment performed better than a pure clinical [34] or biomarker [35] model to predict relevant outcome variables such as AKI progression, recovery of renal function, need for RRT, and death.

We strongly believe that adding biomarker measurement to existing AKI classifications would more accurately confirm both the presence and severity of AKI and allow appropriate stratification and inclusion of patients in well-designed RCTs. This is imperative to correctly assess the real impact of early versus late RRT initiation on patient outcome. Maybe then we will behold the whole elephant!

## Dose of RRT: another factor to take into account?

Theoretically, the prescribed and delivered RRT dose and the timing of RRT initiation must both be considered for controlling uremia in AKI patients [36]. In fact, the dose of RRT may be of prognostic importance if uremic waste product concentration and exposure time become significant. However, “more intensive” RRT has not been shown to improve outcome of critically ill patients with AKI [37]. Studies evaluating the association between RRT dose and outcome also remain difficult to interpret because heterogeneous patient populations were included and different RRT techniques applied [37–40]. Finally, the studies did not address “early vs late” initiation of RRT [36–40].

Consensus is accruing that the delivered RRT dose must be tailored to the needs of an individual patient suffering severe AKI [36]. In addition, investigators will need to carefully consider the RRT dose when evaluating timing of RRT. A paradigm shift in RRT management is evolving and may include an “early” (or delayed) start with a higher (or lower, or initial “higher” followed by “lower”) dose of RRT.In our opinion, RRT strategies should be adapted to particular patient populations. Designing future studies will definitely become more challenging, yet is the only way forward to provide valuable answers on crucial but still unsolved issues in critical care nephrology.

## Conclusions

Because of the substantial differences in study design, patient population, AKI definition, and RRT indication, no conclusive consensus can be generated from existing prospective and retrospective cohort studies, meta-analyses, and the two recent large RCTs which evaluated the relationship between the timing of RRT initiation and patient outcome. There is an urgent need for a cause-specific diagnostic criterion of AKI. We suggest that implementing a sensitive biomarker (panel) on top of current staging classification may allow defining a homogeneous study population to assess the impact of early versus late initiation of RRT on patient outcome.

## Abbreviations

AKI: Acute kidney injury
AKIKI: Artificial Kidney Initiation in Kidney Injury
AKIN: Acute Kidney Injury Network
ELAIN: Early vs Late Initiation of Renal Replacement Therapy in Critically Ill Patients With Acute Kidney Injury
ICU: Intensive care unit
KDIGO: Kidney Disease Improving Global Outcomes
NGAL: neutrophil gelatinase-associated lipocalin
RCT: Randomized controlled trial
RIFLE: Risk, Injury, Failure, Loss, and End-stage
RRT: Renal replacement therapy
